# Editorial: Nutrition and brain: bidirectional link in neuropsychiatry disorders

**DOI:** 10.3389/fnut.2023.1212075

**Published:** 2023-09-26

**Authors:** Lais Bhering Martins, Michael Berk, Adaliene Versiani Matos Ferreira, Antonio Lucio Teixeira

**Affiliations:** ^1^Department of Psychiatry and Behavioral Sciences, University of Texas Health Science Center, Houston, TX, United States; ^2^Deakin University, School of Medicine, The Institute for Mental and Physical Health and Clinical Translation (IMPACT), Barwon Health, Geelong, VIC, Australia; ^3^Department of Nutrition, Nursing School, Universidade Federal de Minas Gerais, Belo Horizonte, Brazil

**Keywords:** nutritional psychiatry, diet, brain, branched-chain amino acids (BCAA), selenium, mental health, fatty acid, psychiatry

Neurological and psychiatric disorders are among the leading causes of disability worldwide (1, 2), causing a huge socioeconomic burden. Despite advances in the field, several therapeutic needs remain unmet, especially regarding prevention strategies. Nutrition-related factors, such as dietary patterns and specific nutrients, can serve as risk factors by influencing pathways implicated in the pathophysiology of neuropsychiatry disorders (3, 4). This emerging area has been named “Nutritional Psychiatry” and has great potential as an adjunctive approach for preventing and treating neuropsychiatric disorders.

This Research Topic aimed to highlight studies on the association between nutrition-related factors and neuropsychiatric disorders, as well as the putative mechanisms underlying this link. It conveys a collection of five original research articles, including one meta-analysis.

Three out of five manuscripts used Mendelian randomization (MR) to assess the apparent causal relationship between specific nutrients (as potentially modifiable risk factors) and neuropsychiatric disorders (as outcomes). MR is a novel analytical method that uses genetic variations as a natural experiment to explore causal relations in observational data. This method is increasingly used because it can address unmeasured confounding, an important limitation of observational studies data analysis (5). Zeng et al. reported that higher levels of adrenic acid (an omega-6 polyunsaturated fatty acid) and eicosapentaenoic acid (EPA, an omega-3 polyunsaturated fatty acid) were associated with a decreased risk of depression, while higher levels of oleic acid (OA, a mono-unsaturated fatty acid) and α-linolenic acid (ALA, a plant-based omega-3) could increase the risk of depression. Qian et al. showed that Alzheimer's disease was related to reduced levels of total branched-chain amino acids (BCAAs), valine, leucine, and isoleucine. Finally, Deng et al. showed the association between circulating selenium levels and decreased risk of schizophrenia. Given the epidemiological evidence that diet quality is a risk factor for depression and other mental health disorders, these studies support the notion that nutritional approaches may contribute to the prevention and/or treatment of neuropsychiatric disorders. In line with that, leveraging big databases (the Global Burden of Diseases, Injuries, and Risk Factors Study 2019 and the United Nations Food and Agriculture Organization), Liang et al. investigated the association between the burden of neuropsychiatric disorders and diet transition (i.e., the changes in diet composition and food production over time) at the national level. They reported an age-related upward pattern in the burden of neuropsychiatric disorders associated with a marked dietary transition, including a reduction in diet quality and food production sustainability. These factors mostly affect the younger generation experiencing a particular increase in neurological and mental disorders disability-adjusted life year (DALY) percent. Several dietary transition factors were also correlated with the increased burden of neuropsychiatric disorders, including increased calorie intake, greater ultra-processed food consumption, alcohol intake, a higher ratio of animal foods to vegetables, and fertilizer use. Besides showing the potential role played by the quality of the diet on the population's mental health, the study suggests that creating sustainable food systems and a healthier food environment can contribute to reducing the burden related to neuropsychiatric conditions beyond the expected environmental and metabolic benefits. These findings have broad implications and may guide public health policies.

Patients with neuropsychiatric disorders have more unhealthy diets and lifestyle habits that, in addition to medication-related side effects, can lead to obesity and other metabolic diseases (3, 4). Importantly, previous studies have shown the association between either a poor-quality diet (6) or obesity (7) with structural brain changes. In this special issue, Dietze et al. demonstrated a reduced fractional anisotropy (FA) with obesity in the genu and splenium of the corpus callosum, middle cerebellar peduncles, anterior thalamic radiation, corticospinal projections, and cerebellum, suggesting that obesity-related brain white matter changes are localized rather than diffuse. However, it remains to be established the mechanisms underlying the link between obesity and detrimental changes in brain structure and function.

While “Nutritional Psychiatry” is still in its early stage of development, seminal studies show that, for example, the Mediterranean diet is efficacious in the management of depression (8). Hence its promises and reach cannot be overstated. This Research Topic provides a snapshot of this exciting field, highlighting the importance of consuming a high-quality diet to potentially prevent neuropsychiatry disorders, and metabolic diseases, such as obesity, that can affect the former ones.

## Author contributions

LM wrote the article. MB, AF, and AT reviewed the text, contributed to the article, and approved the submitted version. All authors contributed to the article and approved the submitted version.
